# Histological assessments of intestinal immuno-morphology of tiger grouper juvenile, *Epinephelus fuscoguttatus*

**DOI:** 10.1186/2193-1801-2-611

**Published:** 2013-11-15

**Authors:** Mohd Firdaus-Nawi, Mohd Zamri-Saad, Nik Yusoff Nik-Haiha, Md Abu Bakar Zuki, Abd Wahid Mohd Effendy

**Affiliations:** Faculty of Veterinary Medicine, Universiti Putra Malaysia, Serdang, Selangor 43400 Malaysia; Marine Finfish Production and Research Centre, FRI Tanjung Demong, Besut, Terengganu 22200 Malaysia; Institute of Marine Biotechnology, Universiti Malaysia Terengganu, Kuala Terengganu, Terengganu 21030 Malaysia

**Keywords:** Tiger grouper, Intestine histological, Assessment, Immune system

## Abstract

Histological assessments on the intestinal morphology and immunity of tiger grouper juveniles, *Epinephelus fuscoguttatus* help in determining the earliest age to start an oral vaccination. This study describes the morphological development of the intestinal immunity of tiger grouper of various ages. Clinically healthy tiger groupers were selected and divided into 4 groups of 20 fish per group. Groups 1, 2, 3 and 4 consisted of juveniles of 30, 60, 90 and 120 days old, respectively. The whole intestine was collected and divided into three regions, the anterior, mid and posterior intestine and fixed in 10% buffered formalin before slides were prepared for microscopic examinations. It was found that the histological structures of the anterior intestine were for absorption of nutrient from digested food particles. The significantly (p < 0.05) higher number and length of the intestinal villi and smaller gap between villi were observed in the anterior intestine, which were structures for absorption. Structures of the posterior intestine were for immunity especially the adaptive immunity with included significantly (p < 0.05) higher numbers of the lymphoid and goblet cells, and significantly (p < 0.05) thicker lamina propria, which were structures for immunity. The mid intestine was the transition structure that involved in both absorption and innate immunity. The results also revealed that leukocytes existed in the lamina propria of 30-days old tiger groupers, an indication that the immune system was present at that particular age.

**Electronic supplementary material:**

The online version of this article (doi:10.1186/2193-1801-2-611) contains supplementary material, which is available to authorized users.

## Introduction

Grouper is a high value cultured marine fish especially in the Southeast Asia countries including Malaysia, Indonesia, Thailand and the Philippines (Liao et al., 2001; Yashiro, 2008). The major cultured species are giant grouper, *Epinephelus lanceolatus* (Yashiro, 2008), tiger grouper, *Epinephelus fuscoguttatus* (Sugama *et al*., 2008), malabar grouper, *Epinephelus malabaricus* (Yashiro, 2008), orange spotted grouper, *Epinephelus coioides* (Toledo et al. 1996), humpback grouper, *Cromileptes altivelis* (Marte, 2003), leopard coralgrouper, *Plectropomus leopardus* (De Silva, 1998) and hybrid grouper, (*Epinephelus lanceolatus* X *Epinephelus fuscoguttatus*). Heemstra and Randall (1993) estimated that 90% of the world’s harvest on marine food is derived from artisanal fisheries, which groupers are the major component. According to Food and Agriculture Organization (FAO) (2013), world aqu of (2880saculture production of groupers was around 6000–7000 tones per annum, valued at about USD60 million and the bulk of this production came from wild seed stock due to lack of reared seed that suffered disease problems.

In Malaysia, tiger grouper, *Epinephelus fuscoguttatus*, also known as brown marbled grouper, is a major cultured marine species. It is considered a popular and high valued aquaculture species and fast growing (Afero et al., 2009). Data from Federal Agricultural Marketing Authority of Malaysia (2013) (FAMA) indicated that grouper species are the major export fish for the year 2010 (MAHA, 2013).

Fish digestive system is slightly differ from mammal. The digestive process has started in the first region that includes the mouth, oral cavity, and pharynx. Then, after food is swallowed it will enter the alimentary canal proper and proceeds via the esophagus to the stomach followed by the intestines (Buddington and Kuz’mina, 2000). The main function of fish intestine is to complete the digestive process, which started in the stomach and also to absorb the nutrients from food (Wilson and Castro, 2011). Other than the digestive function, gut of fish also acts as the first-line barrier against infection. Thus, the mucosal layer of the gut creates physical, chemical and cellular protections against pathogen invasions (Ellis, 2001). The goblet cells and the glandular simple columnar epithelial cells secrete mucus, which contains immunological substances such as glycoprotein (Fletcher and Grant, 1968), cytokines (Lindenstrøm et al. 2003), peptides (Cole et al., 1997), lysozyme (Fernandes et al., 2004), lipoprotein (Concha et al., 2003), complement (Dalmo et al., 1997), lectins (Tsutsui et al., 2005), proteases (Aranishi and Mano, 2000) and antibodies (Cain et al., 2000). These substances provide direct or indirect protection against pathogen (Cain et al., 1996). Furthermore, the intestine of fish contains lymphoid cells that secrete antibodies and involve in phagocytosis. The aim of this study is to describe the histological evaluation on the intestinal immune-morphology of tiger grouper, *Epinephelus fuscoguttatus* juveniles.

## Materials and methods

### Fish and rearing conditions

A total of 80 healthy tiger grouper (*Epeniphelus fuscoguttatus*) juveniles of different ages were used. They were 30, 60, 90 and 120 days old of approximately 1 g ± 0.3 g, 2.5 g ± 0.3 g, 200 g ± 0.3 g and 300 g ± 0.3 g body weight, and of approximately 2.5 cm ± 0.5 cm, 5 cm ± 0.5 cm, 7.5 cm ± 0.5 cm and 10 cm ± 0.5 cm in length, respectively. The selected juveniles were grouped according to the age with 20 fish per group and kept separately in 100-L glass tanks. Fish of less than 30 days old were too small for handling and sampling. All the fish were acclimatized for at least 7 days prior to experiment. Light cycle was held constant with 12 hours of lighting per day. Feeding was *ad libitum* with a local commercial feed while water was continuously aerated. The water temperature, pH, salinity and dissolved oxygen were measured daily using the HQ40d Meter (Hach Company, Loveland, CO). The ammonia, sulfate and nitrites were determined daily using the DR 2800 Portable Spectrophotometer (Hach Company, Loveland, CO). Prior to sampling, five fish from each age group were sacrificed and swab samples from the organs were collected for bacterial and parasitic examinations. This was to ensure that the fish were free from bacterial and parasitic diseases (Firdaus-Nawi et al., 2012).

### Experimental design

At the start of the experiment, the remaining fifteen juvenile tiger groupers from each group were euthanized by an overdose of Ethyl 3-aminobenzoate methanesulfonate (Sigma Aldrich, USA). Post-mortem examination was performed immediately; the entire intestine was removed and was cut into the anterior, mid and posterior portions (Wilson and Castro, 2011) before the intestinal samples were fixed into 10% buffered formaldehyde for at least 24 h. The Animal Care and Use Committee of University Putra Malaysia approved the study protocol.

### Sample preparation and histological analysis

Following 24 h fixation, the samples were prepared for histological examination according to Firdaus-Nawi et al. (2012). The slides were examined under a light microscopy using Nikon NIS-Element D 3.2 Image Analyzer (Nikon Instruments Inc., USA). For each slide, a total of five microscopic fields were examined at 200× magnification to determine the number of villi, the length of villi, the gap between villi, the thickness of lamina propria, the number of lymphoid cells, the number of goblet cells and the thickness of muscular layer. The Nikon NIS-Element D 3.2 Image Analyzer (Nikon Instruments Inc., USA) was used to measure all parameters.

### Statistical analysis

The Statistix 9 (Analytical Software, USA) was employed to analyze the data. The results revealed significance in all pairwise comparison under one-way ANOVA. Significant differences were determined at P < 0.05. Pearson correlation test was used to reveal the correlation between each studied parameters.

## Results

### Number of villi

**Figure 1:**
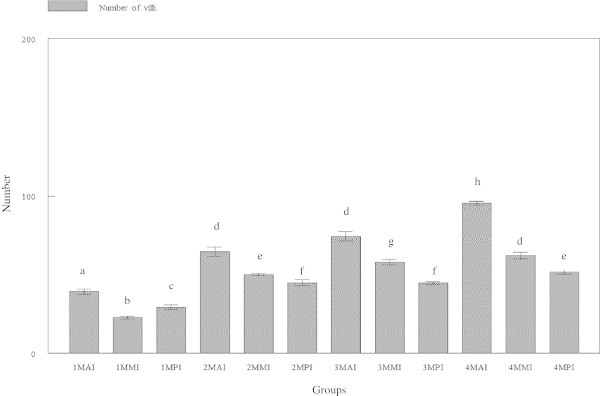
**Number of villi in three regions of tiger grouper intestine; anterior intestine (AI), mid intestine (IM) and posterior intestine (PI), in four experimental ages; 30 days (1 M), 60 days (2 M), 90 days (3 M) and 120 days (4 M).**

There were significant (p < 0.05) differences in the numbers of villi in the three regions of intestine (Figure 1). The average number of villi of anterior intestine was 39.200 ± 6.1435, 64.467 ± 11.077, 74.333 ± 11.709 and 95.533 ± 4.0860 for tiger groupers at 30, 60, 90 and 120 days old, respectively. The average number of villi of mid intestine was 22.533 ± 3.6227, 49.933 ± 2.8149, 57.867 ± 6.8334 and 62.000 ± 8.000 μm at 30, 60, 90 and 120 days old, respectively. The average number of villi of posterior intestine was 29.067 ± 5.1195, 43.333 ± 4.7006, 44.533 ± 3.3989 and 51.467 ± 4.8236 μm, respectively.

**Figure 2:**
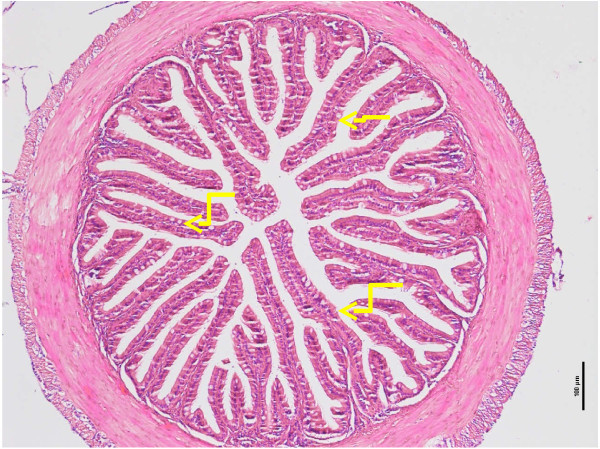
**Cross-section of anterior intestine of 90 days old tiger grouper.** High numbers of villi were observed (arrows) and it is important to make the absorption process very effective (H&E x10).

The number of villi was highest in the anterior region of intestine (Figure 2), followed by the mid region and least in the posterior region, except for the 30 days old tiger grouper that showed that the number of villi was significantly (p < 0.05) higher in the posterior than the mid region. In general, the number of villi showed gradual and significant (p < 0.05) increase with the increasing age of the tiger groupers. However, there was no significant (p > 0.05) increase in the numbers of villi in the mid intestine between 90 and 120 days old tiger grouper. Similar observation was noted for the posterior region of the 60 and 90 days old tiger grouper (Figure 1).

### Length of villi

**Figure 3:**
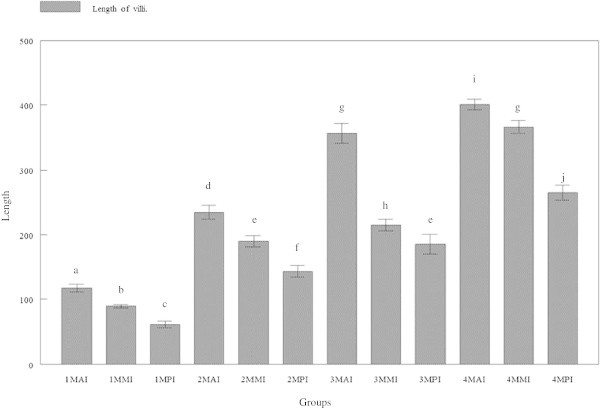
**Length of villi in three regions of tiger grouper intestine; anterior intestine (AI), mid intestine (IM) and posterior intestine (PI), in four experimental ages; 30 days (1 M), 60 days (2 M), 90 days (3 M) and 120 days (4 M).**

**Figure 4:**
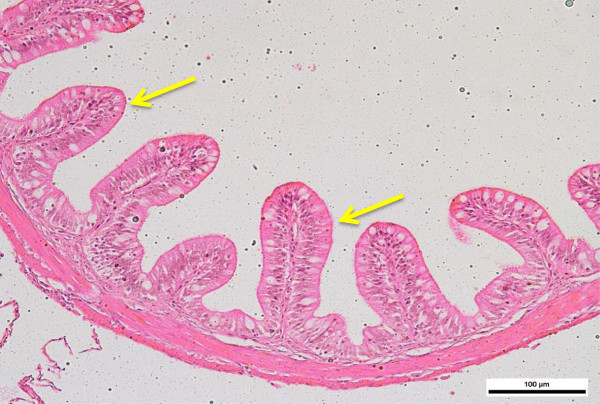
**Cross-section of posterior intestine of 90 days old tiger grouper.** The villi in posterior region is shortest compared to in mid and anterior intestine (arrows) (H&E x20).

Measurements of the length of villi revealed significant (p < 0.05) differences between the intestinal regions of all group tiger groupers (Figure 3). The average length of villi at the anterior intestine was 117.08 ± 23.509, 234.75 ± 42.025, 356.21 ± 59.268 and 400.55 ± 31.133 μm for tiger groupers of 30, 60, 90 and 120 days old, respectively. The average length of villi at the mid intestine was 89.094 ± 10.205, 189.96 ± 34.111, 215.03 ± 34.326 and 365.99 ± 39.110 μm at 30, 60, 90 and 120 days old, respectively. The average number of villi at the posterior intestine was 60.605 ± 20.576, 143.03 ± 35.463, 185.31 ± 59.198 and 265.44 ± 46.064 μm, respectively. For all groups, the length was significantly (p < 0.05) highest in the anterior region, followed by mid region and least in the posterior intestine. Nevertheless, the length showed gradual increase with increasing age of the tiger groupers (Figures 3 and 4).

### Gap between villi

**Figure 5:**
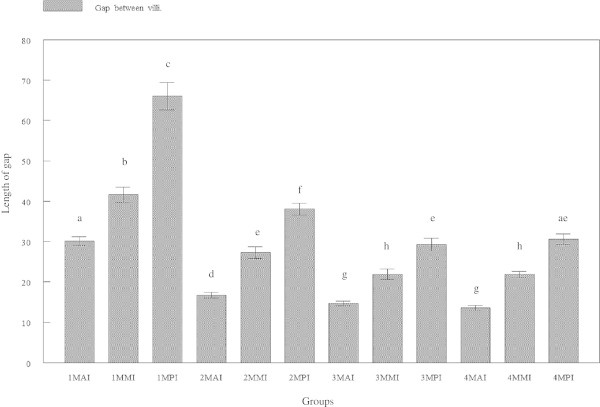
**Cross-section of mid intestine of 120 days old tiger grouper showing intermediately tall villi with lamina propria (arrows) that is thicker than anterior intestine but thinner than the posterior intestine (H & E ×40).**

**Figure 6:**
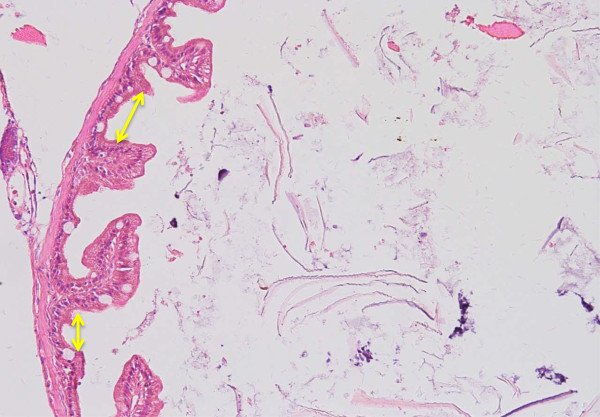
**Cross-section of anterior intestine of 30 days old tiger grouper.** The size of gap between villi in 30 days old tiger grouper is wide (arrows) and reduces when age is increase (H&E x20).

Widest villus gap was observed in the posterior intestine followed by the mid and the smallest gap was in the anterior intestine (Figures 5 and 6). The average gap between villi of the anterior intestine was 30.143 ± 4.0889, 16.756 ± 2.7046, 14.665 ± 2.3584 and 13.606 ± 2.292 μm for tiger grouper at 30, 60, 90 and 120 days old, respectively. The average gap between villi of the mid intestine was 41.690 ± 7.2816, 27.275 ± 5.5747, 21.957 ± 4.9491 and 21.888 ± 2.9411 μm at 30, 60, 90 and 120 days old, respectively. The average gap between villi of the posterior intestine was 66.024 ± 13.096, 38.078 ± 5.7424, 29.258 ± 6.1213 and 30.604 ± 4.9327 μm, respectively.

Villus gap in the posterior region was significantly (p < 0.05) wider than the mid and anterior regions. In all regions of the intestine, the gap was gradually and significantly (p < 0.05) decreasing with increasing age of tiger grouper, except between 30 and 60 days old tiger groupers (Figure 6).

### Thickness of lamina propria

**Figure 7:**
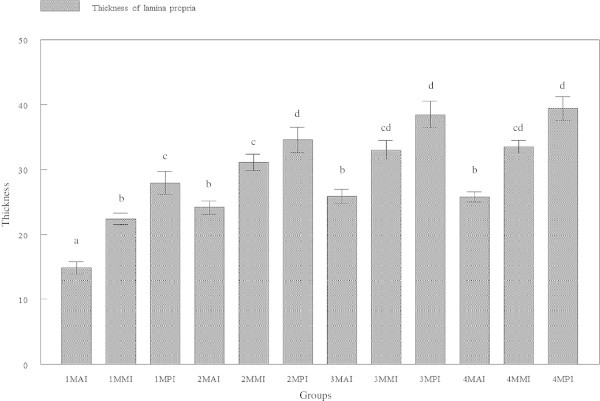
**Cross-section of posterior intestine of 120 days old tiger grouper showing significantly (p < 0.05) high concentration of lymphoid cells (arrows) in the lamina propria (H & E ×40).**

**Figure 8:**
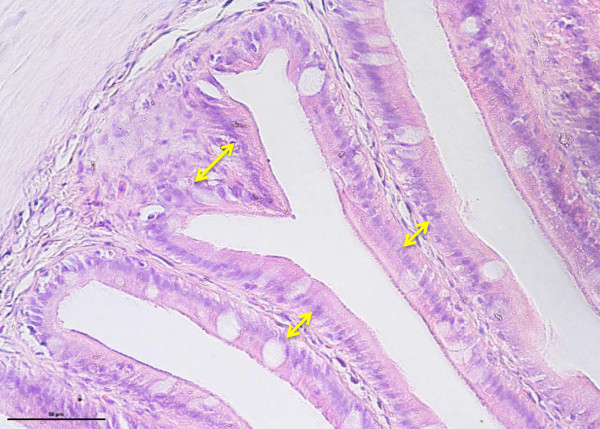
**Cross-section of mid intestine of 120 days old tiger grouper.** The thickness of lamina propria in mid intestine (arrows) are thinner than posterior but thicker than anterior (H&E x40).

Lamina propria in the anterior region was found to be significantly (p < 0.05) thin, followed by the mid region and significantly (p < 0.05) thick at the posterior region. This was observed in all groups of tiger groupers (Figure 7). The average thickness of lamina propria at the anterior intestine was 14.854 ± 3.7412, 24.197 ± 3.9502, 25.889 ± 4.2674 and 25.792 ± 2.8590 μm for tiger groupers at 30, 60, 90 and 120 days old, respectively. The average thickness of lamina propria at the mid intestine was 22.435 ± 3.4695, 31.094 ± 5.0122, 33.002 ± 5.7754 and 33.514 ± 3.8933 μm at 30, 60, 90 and 120 days old, respectively (Figure 8). The average thickness of lamina propria at the posterior intestine was 27.947 ± 6.9433, 34.598 ± 7.6659, 38.509 ± 7.6971 and 39.415 ± 7.1762 μm, respectively.

The thickness of lamina propria in all regions of intestine was observed to gradually increased with age. However, significant (p < 0.05) difference in the thickness of lamina propria was only observed between 30 days old and other groups of 60, 90 and 120 days old tiger grouper in all regions of intestine. The differences in thickness of lamina propria among 60, 90 and 120 days old tiger grouper was found insignificant (p > 0.05) in all regions of the intestine.

### Numbers of lymphoid cells

**Figure 9:**
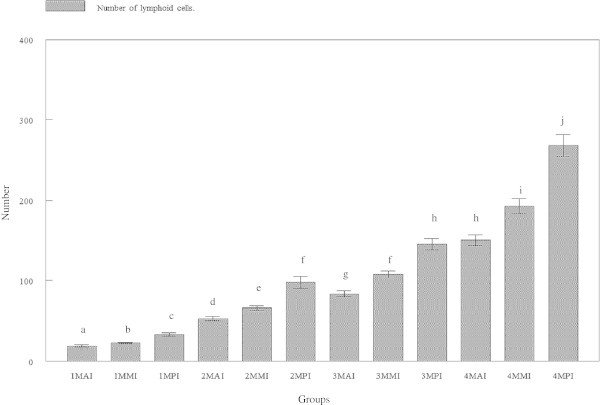
**Number of goblet cells in lamina propria of three regions of tiger grouper intestine; anterior intestine (AI), mid intestine (IM) and posterior intestine (PI), in four experimental ages; 30 days (1 M), 60 days (2 M), 90 days (3 M) and 120 days (4 M).**

**Figure 10:**
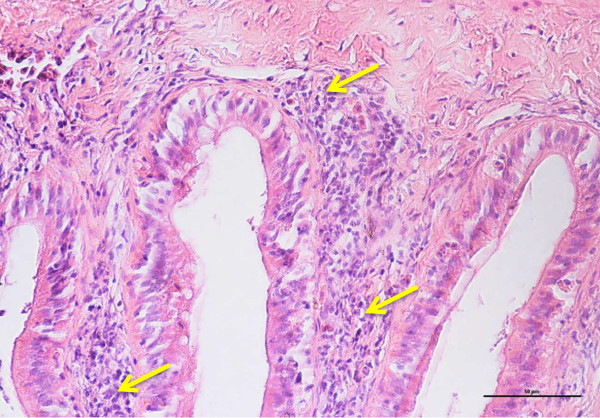
**Cross-section of posterior intestine of 120 days old tiger grouper.** High concentration of lymphoid cells found scattered in lamina propria of posterior intestine (arrows) (H&E x40).

The numbers of lymphoid cells in the lamina propria were found to differ between the different intestinal regions. Significantly (p < 0.05) highest average number of lymphoid cells was found in the posterior intestine followed by the mid and the anterior intestines of all age groups (Figures 9 and 10). The average number of lymphoid cells in the anterior intestine was 18.867 ± 6.1975, 52.867 ± 10.218, 83.400 ± 14.657 and 150.40 ± 25.972 μm for tiger groupers at 30 days, 60 days, 90 days and 120 days old, respectively. The average number of lymphoid cells in the mid intestine was 22.600 ± 3.8508, 65.933 ± 10.807, 107.53 ± 17.635 and 192.67 ± 35.826 μm at 30 days, 60 days, 90 days and 120 days old, respectively. The average number of lymphoid cells in the posterior intestine was 32.867 ± 8.1141, 97.600 ± 28.362, 145.33 ± 26.300 and 268.07 ± 52.138 μm, respectively.

The numbers of lymphoid cells in lamina propria were significantly (p < 0.05) highest in the posterior region, followed by the mid region and least in the anterior region. The number of lymphoid cells in the intestine of 30 days old tiger groupers was significantly (p < 0.05) lower than other studied ages. Similarly, the average number of lymphoid cells in the intestine of 60 days old was significantly (p < 0.05) lower than the 90 days old, which was significantly (p < 0.05) lower than the 120 days old (Figure 9). The number of lymphoid cells in all intestinal regions was observed to gradually increase with age.

### Number of goblet cells

**Figure 11:**
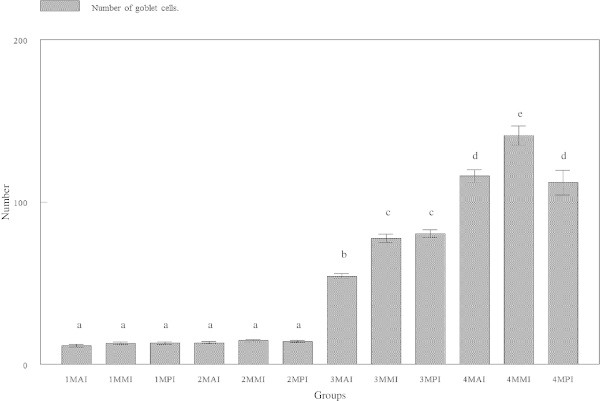
**Number of goblet cells in lamina propria of three regions of tiger grouper intestine; anterior intestine (AI), mid intestine (IM) and posterior intestine (PI), in four experimental ages; 30 days (1M), 60 days (2M), 90 days (3M) and 120 days (4M).**

**Figure 12:**
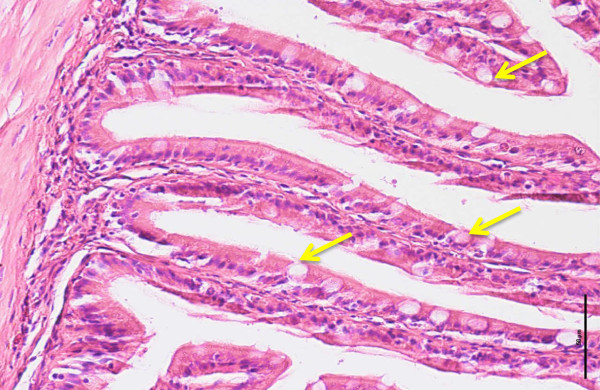
**Cross-section of anterior intestine of 90 days old tiger grouper.** Goblet cells (arrows) found in every regions of intestine (H&E x40).

At the early age, goblet cell counts showed no significant (p > 0.05) differences in all regions of the intestine as observed in the 30 and 60 days old tiger grouper (Figures 11 and 12). The 30 days old groupers had an average number of 11.467 ± 3.2704, 13.000 ± 2.4785 and 13.067 ± 2.5204 cells in the anterior, mid and posterior intestines, respectively compared to 13.200 ± 2.9568, 14.600 ± 2.9228 and 14.133 ± 2.3258 cells in the anterior, mid and posterior intestines of 60 days old groupers, respectively.

However, the number of goblet cells started to significantly (p < 0.05) increased at the age of 90 days old with 54.200 ± 6.2929 cells in the anterior intestine, 77.733 ± 10.117 cells in the mid and 80.600 ± 9.2257 cells in the posterior intestines. The incremental pattern continued in the 120-day old tiger groupers with 116.07 ± 14.733 cells in the anterior, 141.00 ± 22.548 cells in the mid and 112.00 ± 29.653 cells in the posterior intestines. The number of goblet cells in all regions of the 90-day old tiger grouper was significantly (p < 0.05) less than the 120-day old tiger groupers (Figure 11).

### Thickness of intestinal muscle

**Figure 13:**
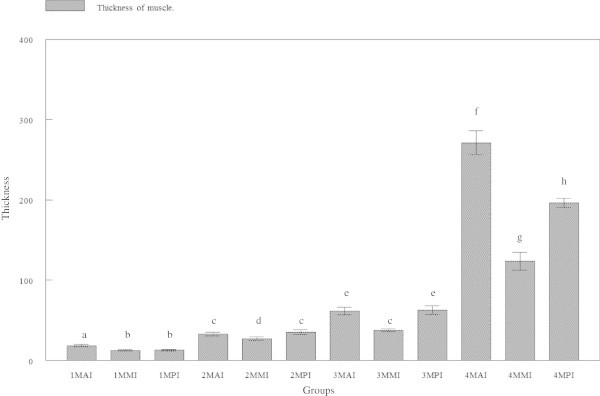
**Thickness of muscle in three regions of tiger grouper intestine; anterior intestine (AI), mid intestine (IM) and posterior intestine (PI), in four experimental ages; 30 days (1M), 60 days (2M), 90 days (3M) and 120 days (4M).**

**Figure 14:**
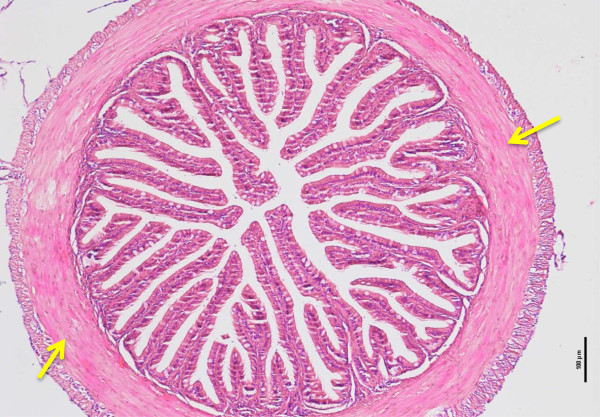
**Cross-section of anterior intestine of 90 days old tiger grouper.** The thickest muscle (arrows) found in anterior region of intestine (H&E x10).

The growth of intestinal muscles was found to be slow in the first three months before drastically increased at the age of 120 days old (Figure 13). At 30 days, the thickness of muscular layer of the anterior intestine was 18.242 ± 5.2238 μm, which was significantly (p < 0.05) thicker than the mid (12.387 ± 3.7875 μm) and posterior (12.637 ± 3.3883 μm) regions (Figure 14). At the ages of 60 and 90 days old, muscle thickness at the anterior regions (32.717 ± 9.5340 μm and 61.287 ± 18.161 μm, respectively) was significantly (p < 0.05) thicker than the mid regions (27.053 ± 7.6956 μm and 37.535 ± 6.8394 μm, respectively), but not significant (p > 0.05) than the posterior regions (35.241 ± 11.660 μm and 62.425 ± 20.315 μm, respectively). However, observation in 120 days old tiger groupers revealed the intestinal muscle to be thickest at the anterior region (271.02 ± 57.174 μm), followed by the posterior region (196.35 ± 23.115 μm) and thinnest in the mid region (123.72 ± 42.411 μm).

Generally, the thickness of intestinal muscle gradually increased with age. The thickness of 30 days old tiger grouper was significantly (p < 0.050 less than the 60 days old, which was significantly (p < 0.05) less than the 90 days old and significantly (p < 0.05) less than the 120 days old tiger groupers.

### Correlations

The number of villi (0.9097), the length of villi (0.9246 μm), the number of lymphoid cells (0.9343), the thickness of lamina propria (0.6621 μm), the number of goblet cells (0.9178) and the thickness of intestinal muscle (0.8262 μm) of the anterior intestine showed direct proportionate to the age of tiger grouper and between each other. Only the gap between villi (-0.7994 μm) was inversely proportional to the age and other parameters (Additional file 1: Table S1). Similar correlations were observed in the mid (Additional file 2: Table S2) and posterior (Additional file 3: Table S3) intestines.

## Discussion

Intestine is a tubular organ where feed from stomach passes through to start an alkaline digestion before the nutrient absorption (Canan et al., 2012). Unlike mammals, there are no distinct separation between small and large intestines of teleost fish (Albrecht et al., 2001). In other word, the intestine is just an undifferentiated tube. Furthermore, the intestine of teleost did not reveal obvious crypts of Lieberkuhn that normally found in mammals. However, Wallace et al. (2005) divided the intestine into three regions namely the anterior, mid and posterior regions.

The intestinal epithelium is a single-cell layer from the type of absorptive columnar cells or enterocytes, which play a role in protecting the fish against harmful agents in lumen. It also functions as a site for nutrient, water and ion uptake (Sundell et al., 2003). In fish, the length of intestine varies and depends on the diet, but basically between 0.4 and 38 times longer than the body length. The amount of plant materials in diet is the major determination factor for intestinal length. Usually, herbivorous fish have longer intestine compared to carnivorous fish (Clements and Raubenheimer, 2006).

This study elaborates the evolution of intestinal tissues and cells of tiger grouper from the age of 30 days until 120 days, which included the villi, lamina propria, lymphoid cells, goblet cells and muscle tissues. Villi are small, finger-like projections that protrude from the epithelial lining of the intestinal wall. Villi are specialized tissues, created to do the absorption in the small intestine as they have a thin wall, about one-cell thick known as enterocyte that enables a shorter diffusion path (Ferraris et al., 1990; Oxley et al., 2007). They also have a large surface area due to their 'loops-like’ shape, for more efficient absorption of nutrients into the blood stream (Bakke et al., 2011). The complete shape and function of villi could be observed in as early as 30 days old tiger grouper for absorption of smaller and solubilized nutrients.

The numbers of villi in the anterior intestine were significantly high since it is the place where mechanically digested feeds or chyme from the stomach started to be absorbed, just like the first filter that filtrate maximum amount of digested feed particles from the stomach. Therefore, the maximum numbers of villi are needed to do the maximum absorption job (Bakke et al., 2011). Similarly, the lengths of villi in anterior intestine were significantly taller. This was to provide more surface area for absorption of nutrient-rich feed particles more efficiently (Nordrum et al., 2000; Bakke et al., 2010). This was in agreement with the conclusion that absorption of nutrients such as protein, carbohydrate and lipid occurred at a faster rate in proximal or anterior regions of intestine (Collie, 1985; Buddington and Diamond 1987; Dabrowski, 1990; Bakke-McKellep et al., 2000; Jutfelt et al., 2007).

The remnants of feed particles that are not absorbed in the anterior intestine then migrate into mid intestine where absorption process continues to occur. Since the amounts of feed particles that migrate toward mid intestine are lesser, the number and length of villi were significantly reduced. Finally, the remaining unabsorbed feed particles and wastes migrate into posterior intestine, waiting to be removed from the body through anus that is located at the end of posterior intestine. Thus, the function of posterior intestine or hindgut is more to immunity than to absorption process (Ezeasor and Stokoe 1981; Buddington and Diamond, 1987). Since the feed demand and consumption increase with age of fish, the number and length of villi keep increasing with age of the tiger grouper.

The lamina propria is a vascularized connective tissue containing nerves and leukocytes, which lies beneath the epithelium and together with the epithelium constitutes the mucosa (Wilson and Castro, 2011). The thickness of lamina propria in anterior intestine was significantly less than the mid intestine but became significantly thicker in the posterior intestine. Therefore, the thickness of lamina propria was inversely proportional with the number of villi. The rapid growth and development of the lamina propria occurred between 30-day and 60-day old groupers when significant increase in the thickness of lamina propria was observed.

This study also revealed that the thickness of lamina propria was related with the concentration of lymphoid cells and the region’s specific function. High concentrations of lymphoid cells were observed in the posterior intestine, and support the conclusion that posterior intestine plays a major role in immunity (Buddington and Diamond, 1987). Similarly, Ezeasor and Stokoe (1981) found high phagocytic activities in the posterior intestine compared to another regions while a study in red tilapia revealed accumulation of lymphoid cells in the lamina propria of posterior intestine following oral vaccination with adjuvanted feed-base vaccine against streptococcosis (Firdaus-Nawi et al., 2012). Due to the immunity functions but less nutrient absorptive capacity (Buddington and Diamond, 1987), posterior intestine requires large numbers of lymphoid cells and the lamina propria should be thick to provide enough space for the lymphoid cells.

The thickness of lamina propria and number lymphoid cells were significantly reduced in the mid intestine. Although the function of mid intestine is not well understood, it was suggested that mid intestine moderately play both absorption and immunity roles. Mid intestine completes the absorption process that was previously done by anterior intestine and starts the immune response prior to the posterior intestine. The presence of lymphoid and goblet cells in intestines of 30-day old tiger grouper indicated that intestinal immunity was present at that particular age. This is in agreement with previous study by Lin et al. (2007) in 19-day old groupers where oral immunization with inactivated nervous necrosis virus (NNV) provided good protection after challenge with live NNV.

Empty space between two villi is called villous gap that appears to be influenced by the number of villi. Size of the gap in region that was packed with villi, such as the anterior intestine, was narrower than the size in mid and posterior intestines where the number of villi was less. The function of the villous gap is to provide spaces for food particles to be absorbed by the villi. Therefore, the gap in 30 days old tiger grouper was significantly larger due to the feeding behavior where young tiger grouper usually consume less food.

Goblet cells are glandular simple columnar epithelial cells and the major mucous cell type in the intestine of fishes (Wilson and Castro, 2011). The main function of goblet cells is to secrete mucin that dissolves in water to form mucus, a clear, colorless and slimy substance that creates a layer to coat the wall of intestine (Kim and Samuel, 2010). Besides function as a lubricant for smooth movement of feed particles, mucus also provides innate host defense by acting as a first line of immunity against invasion of harmful pathogen (Kim and Samuel, 2010). Intestinal mucus secreted in fish contains antibody (Grabowski et al., 2004; Firdaus-Nawi et al., 2012), lysozyme (Lie et al., 1989), glycoprotein (Shephard, 1994), complement components, lectins and some antimicrobial agents (Ingram, 1980; Alexander and Ingram, 1992). In this study, goblet cells were found scattered among the epithelial lining in all three regions of the intestine, but showed no significant difference in numbers until 90 days old. Between 90 and 120 days old, the numbers of goblet cells in mid intestine were dramatically increased, suggesting the important non-specific or innate immunity role played by the region. Therefore, the innate immune system in the mid intestine detects and prevents the presence of invasive pathogen prior to stimulation of the specific immune system in the posterior intestine.

Teleost intestines are lack of muscularis mucosa that divides lamina propria from sub-mucosa (Wallace et al., 2005). The development of intestinal muscle can be attributed to the feeding regime. From birth to the age of 40 days old, tiger grouper were fed with live feed since their digestion system; particularly due to intestinal muscle which less developed. This makes oral vaccination via live feed possible for juvenile groupers At the age of 40 days old and above, they were fed with larvae pellet where the intestinal muscle became thicker as observed in the 60 and 90 days old tiger groupers. Large-sized pellet was introduced to the tiger grouper at the age of 100 days, thus the thickest muscular layer was observed in tiger grouper at age 120 days old.

## Conclusion

From the study, we have found the intestinal immunity in tiger grouper is existed as early as thirty days old age, and every region of intestine have different roles either in food processing or immunity. Since the intestinal immunity is existed at the age of thirty days old, vaccination could be conducted at that particular age. However, vaccination at 60-day old is expected to stimulate stronger immune response since the immune cells were well establsihed.

## Electronic supplementary material

Additional file 1: Table S1: Correlation (Pearson) value between each studied parameters in anterior intestine, age of tiger grouper (AGE), gap between villi (GBV), thickness of lamina propria (TLP), length of villi (LOV), number of goblet cells (NGC), number of villi (NOV), number of lymphoid cells (NLC) and thickness of muscle (TOM). (DOC 33 KB)

Additional file 2: Table S2: Correlation (Pearson) value between each studied parameters in mid intestine, age of tiger grouper (AGE), gap between villi (GBV), thickness of lamina propria (TLP), length of villi (LOV), number of goblet cells (NGC), number of villi (NOV), number of lymphoid cells (NLC) and thickness of muscle (TOM). (DOC 34 KB)

Additional file 3: Table S3: Correlation (Pearson) value between each studied parameters in posterior intestine, age of tiger grouper (AGE), gap between villi (GBV), thickness of lamina propria (TLP), length of villi (LOV), number of goblet cells (NGC), number of villi (NOV), number of lymphoid cells (NLC) and thickness of muscle (TOM). (DOC 33 KB)
