# Supplementary material for: Histological assessments of intestinal immuno-morphology of tiger grouper juvenile, Epinephelus fuscoguttatus
Source: Springerplus. 2013 Nov 15;2:611. doi: 10.1186/2193-1801-2-611 (PMC3858595; doi:10.1186/2193-1801-2-611)
Supplement: Supplementary file 2 — Additional file 2: Table S2: Correlation (Pearson) value between each studied parameters in mid intestine, age of tiger grouper (AGE), gap between villi (GBV), thickness of lamina propria (TLP), length of villi (LOV), number of goblet cells (NGC), number of villi (NOV), number of lymphoid cells (NLC) and thickness of muscle (TOM). (DOC 34 KB) [file 40064_2013_683_MOESM2_ESM.doc]

**Additional file 2:Table S2.** Correlation (Pearson) value between each studied parameters in mid intestine, age of tiger grouper (AGE), gap between villi (GBV), thickness of lamina propria (TLP), length of villi (LOV), number of goblet cells (NGC), number of villi (NOV), number of lymphoid cells (NLC) and thickness of muscle (TOM).

|  | **AGE** | **GBV** | **TLP** | **LOV** | **NGC** | **NOV** | **NLC** |
| --- | --- | --- | --- | --- | --- | --- | --- |
| **GBV** | -0.7513 |  |  |  |  |  |  |
| **TLP** | 0.6218 | -0.5156 |  |  |  |  |  |
| **LOV** | 0.9229 | -0.6684 | 0.6212 |  |  |  |  |
| **NGC** | 0.9236 | -0.5759 | 0.4463 | 0.8440 |  |  |  |
| **NOV** | 0.8642 | -0.7648 | 0.7238 | 0.7796 | 0.6726 |  |  |
| **NLC** | 0.9361 | -0.6293 | 0.5691 | 0.9161 | 0.8938 | 0.7788 |  |
| **TOM** | 0.7978 | -0.4895 | 0.4251 | 0.8537 | 0.8358 | 0.5689 | 0.8119 |
