# Supplementary material for: Histological assessments of intestinal immuno-morphology of tiger grouper juvenile, Epinephelus fuscoguttatus
Source: Springerplus. 2013 Nov 15;2:611. doi: 10.1186/2193-1801-2-611 (PMC3858595; doi:10.1186/2193-1801-2-611)
Supplement: Supplementary file 3 — Additional file 3: Table S3: Correlation (Pearson) value between each studied parameters in posterior intestine, age of tiger grouper (AGE), gap between villi (GBV), thickness of lamina propria (TLP), length of villi (LOV), number of goblet cells (NGC), number of villi (NOV), number of lymphoid cells (NLC) and thickness of muscle (TOM). (DOC 33 KB) [file 40064_2013_683_MOESM3_ESM.doc]

Additional file 3: **Table S3.** Correlation (Pearson) value between each studied parameters in posterior intestine, age of tiger grouper (AGE), gap between villi (GBV), thickness of lamina propria (TLP), length of villi (LOV), number of goblet cells (NGC), number of villi (NOV), number of lymphoid cells (NLC) and thickness of muscle (TOM).

|  | **AGE** | **GBV** | **TLP** | **LOV** | **NGC** | **NOV** | **NLC** |
| --- | --- | --- | --- | --- | --- | --- | --- |
| **GBV** | -0.7658 |  |  |  |  |  |  |
| **TLP** | 0.5076 | -0.4155 |  |  |  |  |  |
| **LOV** | 0.8670 | -0.6551 | 0.4905 |  |  |  |  |
| **NGC** | 0.8946 | -0.6198 | 0.4050 | 0.7301 |  |  |  |
| **NOV** | 0.8263 | -0.7202 | 0.4096 | 0.7267 | 0.6826 |  |  |
| **NLC** | 0.9185 | -0.6422 | 0.4810 | 0.8021 | 0.8422 | 0.7764 |  |
| **TOM** | 0.8847 | -0.5342 | 0.3689 | 0.7736 | 0.7982 | 0.6973 | 0.8817 |
